# Comparison of Organoids from Menstrual Fluid and Hormone-Treated Endometrium: Novel Tools for Gynecological Research

**DOI:** 10.3390/jpm11121314

**Published:** 2021-12-06

**Authors:** Caitlin E. Filby, Katherine A. Wyatt, Sally Mortlock, Fiona L. Cousins, Brett McKinnon, Kate E. Tyson, Grant W. Montgomery, Caroline E. Gargett

**Affiliations:** 1 The Ritchie Centre, Hudson Institute of Medical Research, Clayton, VIC 3168, Australia; Katherine.Wyatt@hudson.org.au (K.A.W.); fiona.cousins@hudson.org.au (F.L.C.); Caroline.Gargett@hudson.org.au (C.E.G.); 2 Obstetrics and Gynaecology, Monash University, Clayton, VIC 3168, Australia; Kate.Tyson@monashhealth.org; 3 Institute for Molecular Biosciences, The University of Queensland, Brisbane, QLD 4072, Australia; s.mortlock@imb.uq.edu.au (S.M.); b.mckinnon@imb.uq.edu.au (B.M.); g.montgomery1@uq.edu.au (G.W.M.); 4 Gynaecological Endoscopy and Endometriosis Surgery, Monash Health, Clayton, VIC 3168, Australia

**Keywords:** endometrial organoids, menstrual fluid, endometriosis, adenomyosis, disease modelling, hormones, human, gynecology

## Abstract

Endometrial organoids (EMO) are an important tool for gynecological research but have been limited by generation from (1) invasively acquired tissues and thus advanced disease states and (2) from women who are not taking hormones, thus excluding 50% of the female reproductive-aged population. We sought to overcome these limitations by generating organoids from (1) menstrual fluid (MF; MFO) using a method that enables the concurrent isolation of menstrual fluid supernatant, stromal cells, and leukocytes and (2) from biopsies and hysterectomy samples from women taking hormonal medication (EMO-H). MF was collected in a menstrual cup for 4–6 h on day 2 of menstruation. Biopsies and hysterectomies were obtained during laparoscopic surgery. Organoids were generated from all sample types, with MFO and EMO-H showing similar cell proliferation rates, proportion and localization of the endometrial basalis epithelial marker, Stage Specific Embryonic Antigen-1 (SSEA-1), and gene expression profiles. Organoids from different disease states showed the moderate clustering of epithelial secretory and androgen receptor signaling genes. Thus, MFO and EMO-H are novel organoids that share similar features to EMO but with the advantage of (1) MFO being obtained non-invasively and (2) EMO-H being obtained from 50% of the women who are not currently being studied through standard methods. Thus, MFO and EMO-H are likely to prove to be invaluable tools for gynecological research, enabling the population-wide assessment of endometrial health and personalized medicine.

## 1. Introduction

The diagnosis and study of gynecological disease currently utilizes endometrial biopsies that are often acquired under general anesthesia. The histopathological analysis of biopsies can confirm the presence of polyps and can diagnose hyperplasia and endometrial cancer. Endometrial biopsies do not provide information about the function of endometrial cells, which are important for fertility and are implicated in diseases such as endometriosis, adenomyosis, Asherman’s syndrome, and infertility. Until recently, the extensive study of endometrial cell behavior was limited to the stromal fraction and endometrial mesenchymal stem cells (eMSC), which was made possible by the identification and prospective isolation of eMSC [1]. While colony forming assays allowed the identification of endometrial epithelial stem/progenitor cells [2,3,4], the study of the endometrial epithelium was curtailed by an inability to maintain those cells in long-term culture. Recently, the generation of organoids from endometrial tissue [5,6,7] has enabled the long-term culture of endometrial epithelial cells, aiding our understanding of endometriosis [6] and fertility [8]. However, these organoids are (1) derived invasively from endometrial biopsies, hysterectomy tissue, and excised endometriosis lesions and thus require surgical intervention and often represent advanced disease states and (2) are derived from women who not taking hormones and thus may not capture the biology of 50% population that is of reproductive age (d).

Menstrual fluid represents a novel biofluid with the potential for diagnosis and disease modeling [9]. We have shown that a range of menstrual fluid cells and proteins have minimal variation from cycle to cycle [9] and that endometrial stem and progenitor cells can be found in menstrual fluid and the pelvic cavity and therefore may play a role in endometriosis [9,10]. Therefore, we proposed that menstrual fluid could be a non-invasive source of endometrial organoids that may enable the population-wide study of early disease.

Current endometrial organoids have been derived from the endometrium of women who are not taking hormonal contraception, despite the fact that a large proportion of young women take some form of hormone for menstrual cycle manipulation. We have shown that endometrium contains epithelial stem/progenitor cells, including in the samples from post-menopausal endometrium [11], that are reactivated upon the administration of estrogen [3] and in hormone-treated endometrium [12]. Therefore, we proposed that the endometrial tissue from women taking hormones could be a source of endometrial organoids that may enable the population-wide study of endometrial epithelial cell behavior in spite of exogenous hormone status.

In this study, we aimed to compare the endometrial organoids that were derived from endometrial biopsies or hysterectomies (EMO) to those derived from menstrual fluid (MFO) and from endometrial tissue from women taking hormonal medication (EMO-H).

## 2. Materials and Methods

### 2.1. Ethics Approval and Consent to Participate

This study was conducted in accordance with the Declaration of Helsinki. All human tissues were collected following ethical approval from Monash Health and Monash University Human Research Ethics Committees (HREC). Menstrual fluid (HREC 20-0000-159A, 09349B, and 01067B) and endometrial biopsies and hysterectomy tissues (HREC 20-0000-159A, 08078B, and 01067B) were collected with approval and written informed consent from all participants. Participant information and demographic data were collected with each sample.

### 2.2. Human Samples

Human menstrual fluid samples (n = 2) were obtained from pre-menopausal women with normal menstrual bleeding or endometriosis (Supplementary Table S1). The exclusion criterion for participation was the use of hormonal contraceptives and/or other exogenous hormonal treatment within the last 3 months. Menstrual fluid samples were collected using a silicon menstrual cup (Lunette, Juupajoki, Finland), which was worn by the participants for 4–6 h on day 2 of menstruation, as previously described [9]. Samples were transferred into 50 mL polypropylene tubes by the participants, transported at 4 °C, and received by laboratory research staff within 2 h of collection. Menstrual fluid supernatants were collected by centrifugation and were frozen immediately (<2 h from collection time). The cellular fraction was processed immediately or within 24 h of collection.

Human endometrial biopsies (n = 4) and hysterectomy (n = 1) samples were obtained from pre-menopausal women undergoing laparoscopy for menorrhagia, endometriosis, adenomyosis, fibroids, infertility, or pelvic pain (Supplementary Table S1). Biopsies were obtained via dilatation and curettage prior to the insertion of laparoscopy ports, and hysterectomy samples were obtained after the laparoscopic removal of the uterus. The use of hormonal contraceptives and/or other exogenous hormonal treatment within the last 3 months was an exclusion criterion for participation in the EMO group and an inclusion criterion for participation in the EMO-H group. Endometrial biopsy and hysterectomy tissues were stored in Collection Medium (DMEM/F12 with HEPES, 1% Antibiotics, 5% neonatal calf serum (NCS); Invitrogen, Carlsbad, CA, USA) at 4 °C and were processed between 2–18 h. Hospital pathology records were used to confirm disease state, hormone state, and menstrual cycle stage (Supplementary Table S1).

### 2.3. Generation of Organoids

#### 2.3.1. Generation of EMO and EMO-H

Human endometrial biopsy and hysterectomy tissues were digested as previously described [2,13]. For the hysterectomy samples, the endometrial tissue was scraped from the myometrial layer. Scraped endometrium and the inner 1 mm of the myometrium, which contains the junctional zone and the basalis endometrial glands were digested. Briefly, the endometrium (and inner 1mm myometrium containing basalis epithelial glands for hysterectomy samples) was fragmented into 1 mm^3^ pieces using small curved scissors before enzymatic (Worthington Collagenase 1, LS004197) trituration and mechanical (Ratek Orbital Shaker, 25 min) digestion with microscopic monitoring until the glandular fragments could be distinguished from the single stromal cells. The sample was filtered (Falcon 40 µm cell strainer FAL352340); the filtrate (mostly single stromal cells) underwent density gradient (Ficoll–Paque) centrifugation (438 rcf, 20 °C, 15 min, no brake) before cryopreservation, while the remaining undigested tissue (mostly epithelial gland fragments) underwent a second digestion with Collagenase 2 (LS004176, Worthington) for approximately 10min or until clusters of 5–10 epithelial cells remained. Epithelial gland clusters were filtered from single cells and were seeded at approximately 7000 clusters per well in Matrigel^®^, as described previously [5,6,7].

#### 2.3.2. Generation of MFO

The human MF samples were digested as previously described [9] with the exception that the collagenase 2 digestion was halted when clusters of 5–10 epithelial cells could be seen microscopically (usually after 10 min). Clusters were then enriched for by epithelial cell adhesion molecule (EpCAM) magnetic beading (4 beads/cell, CELLection Epithelial Enrichment kit, 162.03, Invitrogen) before seeding at 7000 clusters per well in Matrigel^®^, as described previously [5,6,7]. Excess clusters were cryopreserved (30% fetal bovine serum (FBS), 10% Dimethyl sulfoxide (DMSO), Advanced DMEM:F12, 10 uM Rock Inhibitor) for the subsequent generation of MFO upon thawing. MF supernatant, CD45^+^ leukocytes, Sushi domain-containing protein 2^+^ (SUSD2^+^) eMSC, and SUSD2^−^ stromal cells were isolated as described [1,9] and were cryopreserved (90% FBS, 10% DMSO) for subsequent experiments.

#### 2.3.3. Maintenance of EMO, MFO and EMO-H

Media were changed every 2–3 days [5], and organoids were passaged every 7–10 days, as described previously [5,6,7].

### 2.4. Proliferation Assay

The proliferation of the organoids were determined by the PrestoBlue Cell Viability Reagent (A13261, Invitrogen) assay, which was conducted on passage 3 organoids at day 0, 1, and 7 after seeding. Briefly, EMO media was replaced with 200 µL of basal medium containing 1 × PrestoBlue for 1 h before removing the Presto Blue and reading the fluorescence (excitation: 540 nm; emission: 590 nm) of the supernatant on a plate fluorometer (SpectraMax i3 Platform). Fresh EMO media was added to the wells for subsequent culture and measurement at later timepoints. Fluorescence was corrected for background-using wells that were devoid of cells and was calculated as the fold change of the reading at day 1.

### 2.5. Flow Cytometric Analysis

The expression of cell surface markers EpCAM, Neural-cadherin (N-cadherin, NCAD, a marker of endometrial epithelial progenitor cells, eEPC), and Stage Specific Embryonic Antigen-1 (SSEA-1, a putative eEPC marker) were determined by flow cytometry as previously described [9]. Briefly, Passage 3 day 7 organoids were dissociated to clusters mechanically [7] and subsequently to single cells with TryplE Express Enzyme (12604-021, Invitrogen). Cell suspensions were blocked (FcR; 2 μL/10^6^ cells, Miltenyi Biotec and Rat IgG; 4.4 μg/10^6^ cells, Jackson ImmunoResearch, PA USA) on ice for 10 min and were incubated with antibody-fluorochrome conjugates (Supplementary Table S2) for 25 min in the dark at 4 °C. Stained cells were washed, resuspended in 2% FBS/phosphate-buffered saline (PBS), and stained with propidium iodide (PI; 31.25 ng/mL; BD Biosciences). A Compensation Plus Particle Set (60 µL/test; BD Biosciences) was utilised for single-stain controls. Cells were analysed (LSR-Fortessa X-20 flow cytometer; FACS DIVA software, BD Bioscience) and compensated using FlowJo Software (Version 10, FlowJo, LLC). Electronic gating using single-stain and fluorescent minus one (FMO) control tubes and the percentage of viable endometrial cells was determined for EpCAM^+^, EpCAM^+^NCAD^+^, EpCAM^+^SSEA-1^+^, and EpCAM^+^NCAD^+^SSEA-1^+^ cells, as described previously [9].

### 2.6. Immunofluorescence

The localisation of cell surface marker SSEA-1 was determined on wholemount Passage 3 day 7 organoids fixed with paraformaldehyde (30 min, room temperature), washed with PBS, and stored in PBS at 4 °C until staining using an adapted method [14]. All of the washes and incubations were performed with gentle horizontal agitation (30–50 rpm), and the organoids were allowed to settle for 3min prior to the removal of wash/supernatant. Precoated tips were used at each step to avoid organoid loss.

Briefly, the organoids were permeabilised and blocked for 1 hr at room temperature (PBS containing 0.5% Triton X-100, 1% bovine serum albumin (BSA), and 1% goat serum) and were then washed twice (1 mL PBS-BSA 0.1%) for 3 min each. The organoids were then incubated with mouse anti-SSEA-1 (Merck MAB4301, 1:50) or IgM isotype control (Caltag MGM00) in PBS containing 0.05% Triton X-100, 0.1% BSA, and 0.1% goat serum, for 3 days at 4 °C. The primary antibody was removed, and the organoids were washed in 1 mL PBS-BSA 0.1% (5 × 3 min then 2 × 15 min). The organoids were then incubated at 4 °C in the secondary antibody (goat anti mouse AF488, 1:250, Abcam Ab11001) in PBS containing 0.05% Triton X-100, 0.1% BSA, and 0.1% goat serum for 24 hr in the dark. Nuclei staining was performed by adding 500 µL of Hoechst 33342 (0.4 µM in stock solution PBS containing 0.05% Triton X-100, 0.1% BSA, and 0.1% goat serum) and incubating for 2 h at 4 °C. The secondary antibody/Hoechst solution was removed, and the organoids were washed in 1mL PBS-BSA 0.1% (5 × 3 min then 2 × 15 min). The organoids were stored in PBS at 4 °C until the day of imaging, when they were transferred in 50 µL PBS per well into a 96-well flat bottom plate.

Confocal images were acquired with using an FV1200 Olympus confocal microscope. Z stacks were acquired at X20 at 2 µm intervals for a minimum of 15 images. All 2D images were acquired at X20 + the zoom function. Images and Z stacks were adjusted linearly for brightness/contrast using ImageJ.

### 2.7. RNA Sequencing

Gene expression was determined on Passage 3 organoids grown to day 7 and snap frozen until RNA extraction (Qiagen microRNAeasy, 74004). RNA samples with an RIN scores > 8.5 were selected for library generation (Trio RNA-Seq library preparation, Tecan) and sequencing (Illumina next Seq550, 150 bp PE). Read quality was checked using FastQC v0.11.7 [15] and MultiQC v1.6 [16]. The first 5 bp on the 5′ end of each read, along with low quality reads, were trimmed using Trimmomatic v0.39 [17]. HISAT2 v2.2.1 [18] was used to map trimmed reads to the Ensembl Homo sapiens GRCh38 release 84 reference genome followed by the assembly of the known transcripts (Ensembl Homo sapiens CRCh38 release 91 reference assembly) and counting using StringTie v.2.1.6 [19,20]. Transcript counts for each sample were expressed in “fragments per kilobase of transcript per million mapped reads” units, and the raw gene counts were extracted using a Python script that was provided by StringTie.

### 2.8. Normalisation of Gene Counts

The genes were filtered to remove those that were expressed at a low level (genes with counts per million (CPM) <0.48 (~10 counts) and that were expressed in <2 samples) and to remove the mitochondrial genes (mtGenes) that remained after library generation. Raw counts were normalised using the Trimmed Mean of M (TMM) [21] method in edgeR R package v.3.34.1 [21], which corrects for composition bias and library size. Post normalisation counts were converted to CPM.

### 2.9. Gene Expression Analysis

Principle component analysis (PCA) was performed on normalised counts using the “prcomp” function in R. We investigated variation in the expression of the genes in pathways of interest from the GO Biological Pathways and those that were previously defined by Cindrova-Davies et al. between organoids using hierarchical clustering and heat maps generated using the “Heatmap” function in the ComplexHeatmap R package.

### 2.10. Statistics

The proportion of cells determined by flow cytometry are reported as mean ± SEM (95% CI). Descriptive statistics were used due to low sample numbers.

## 3. Results

### 3.1. Organoids Can Be Generated from MF and Endometrial Tissue from Women Taking Hormonal Medication

**Figure 1:**
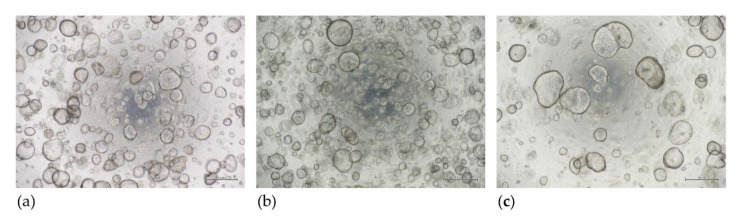
Novel endometrial organoids, passage 3; day 7. Menstrual fluid organoids (MFO) (**a**) and endometrial organoids derived from women taking hormonal medication (EMO-H) (**b**) can be generated and can recapitulate standard EMO (**c**). Bar 500 µm.

We found that organoids could be generated from the menstrual fluid and from endometrial tissue of women taking hormonal contraceptives with high efficiency (80–100%). Organoids could be cultured for up to 8 (MFO) or 5 (EMO-H) passages as a minimum (further passaging is ongoing). The amount of glandular material that was available for organoid generation was often lower in the menstrual fluid (MFO) and endometrium from the women who were on hormones (EMO-H) compared to the endometrial biopsies from the women who were not on hormones. Once the organoids were generated, the number and size of the organoids appeared to be similar for MFO compared to EMO; however, the number of organoids appeared to be greater in the EMO-H samples (Figure 1).

### 3.2. MFO and EMO-H Show Similar Proliferation, Cell Surface Phenotype and eEPC Marker Localisation as Standard EMO

**Figure 2:**
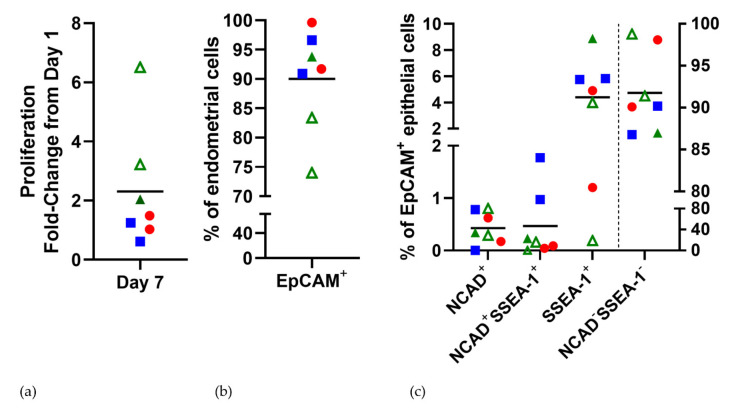
MFO and EMO-H show similar proliferation rates (**a**) and cell surface marker expression (**b**,**c**) to EMO. Proliferation rates are expressed as the fold changes on day 1. ▪ EMO; ● MFO; green triangles EMO−H; 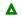 open EMO−OCP; 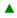 closed EMO-Mirena^®^.

The organoids from all three sources showed a similar proliferation rate on day 7 of culture (fold change of 2.31 ± 0.77 (0.43–4.19)) (Figure 2a). The proliferation of EMO, MFO, and EMO-H were not significantly different from one another. However, there was a trend for a greater rate of cell proliferation of the EMO-H from women taking OCP (open green triangles; Figure 2b) compared to EMO (red circles), MFO (blue squares), and EMO-H from women with a Mirena^®^ IUD (solid green triangle).

**Figure 3:**
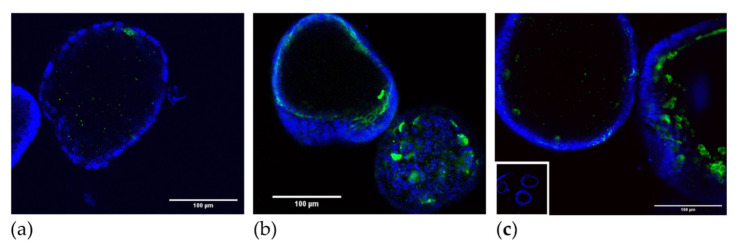
Localization of SSEA-1 in MFO (**a**), EMO-H (**b**), and (**c**) EMO. Inset: IgM control. Bar 100 µm.

The cell surface marker profiling of mature organoids (day 7) demonstrated that almost all cells were EpCAM^+^ epithelial cells (90.0 ± 3.29% (82.0–98.0); Figure 1b). Rare cells were EpCAM^+^NCAD^+^ (0.43 ± 0.12% (0.14–0.72)) and or double positive for the endometrial epithelial stem/progenitor markers EpCAM^+^NCAD^+^SSEA-1^+^ (0.47 ± 0.25% (1.66–7.13)). In comparison, the organoids contained a greater proportion of EpCAM^+^SSEA-1^+^ (4.39 ± 2.96% (1.66–7.13)). The remaining cells were EpCAM^+^NCAD^−^SSEA-1^−^ mature epithelial cells (91.8 ± 1.84% (87.3–96.3); n = 7), and there was also some variation in the expression level between different patient-derived lines that did not correlate with proliferation rates. The organoids from all of the sources showed the localization of SSEA-1 (Figure 3), often at the luminal surface, albeit with varying frequency.

### 3.3. MFO, EMO-H and EMO Have Overlapping Gene Signatures

**Figure 4:**
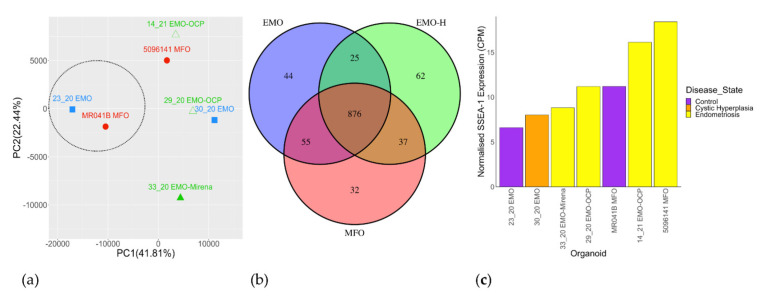
Gene expression profile of MFO, EMO-H, and EMO determined by RNAseq. PCA plot (**a**) of top 1000 genes expressed by MFO, EMO−H, and EMO. MFO (red), EMO (blue), and EMO−H (green) did not cluster separately. Controls (dotted) clustered separately on PC1 only. Venn diagram (**b**) showing the majority of the top 1000 expressed genes were shared by MFO, EMO-H, and EMO. Normalised SSEA-1 expression (**c**) was increased in two of the four women with endometriosis.

Gene expression analysis revealed no obvious clustering of organoid types (Figure 4a). There was a moderate degree of control clustering compared to endometriosis, adenomyosis, and cystic hyperplasia. One sample, 33_20, was distinct from all of the others in terms of both PCA and participant characteristics (likely due to being a hysterectomy sample containing basalis glands, Mirena^®^). Of the top 1000 most highly expressed genes, 876 were shared between all of the organoid types (Figure 4b). The levels of normalized SSEA-1 mRNA levels (Figure 4c) were variable, particularly for participants with endometriosis, two of which (14_21, 5096141) showed higher SSEA-1 compared to the other five participants (including two with endometriosis, 33_20 and 29_20). There was no correlation between the normalized SSEA-1 mRNA levels (Figure 4c) and the % SSEA-1^+^ cells (Figure 3c). An average of 25 m reads aligned for all samples.

**Figure 5:**
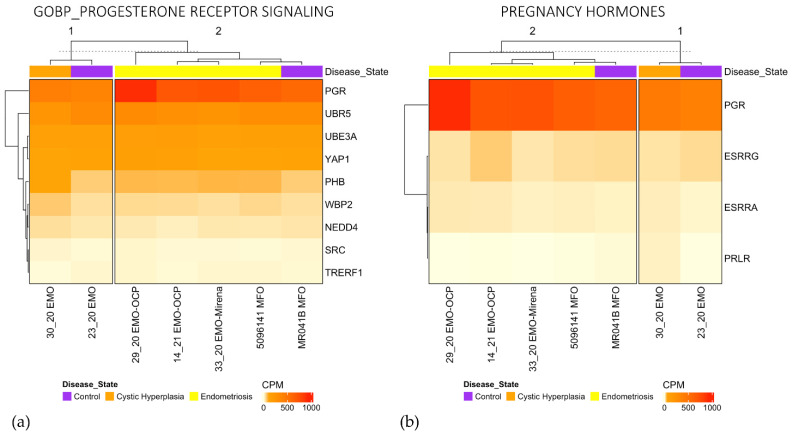
Heatmaps for Progesterone Receptor Signaling (**a**) and Pregnancy Hormones (**b**) show clustering of EMO.

PCA did not show the clear separation between organoid types; however, the analysis of individual gene sets showed that EMO clustered moderately separately from EMO-H and MFO for Progesterone Receptor Signaling and Pregnancy Hormones (Figure 5a,b). MFO clustered together (Supplementary Figure S2a) for Response to Progesterone, but they were not substantially different to one EMO.

### 3.4. EMO-H Do Not Exhibit a Significantly Different Gene Expression Profile Compared to Organoids from Untreated Women

Hormonal treatments suppress circulating estrogen concentrations and can have a significant influence on endometrial biology, including both target and non-target receptors. As such, we used curated gene sets from the GO Biological Pathways to determine the influence on response, signaling pathway, and metabolism of the hormone receptors that are associated with organoids that are derived from the endometrium and menstrual fluid or those that are derived from the women who were taking hormonal treatments.

**Figure 6:**
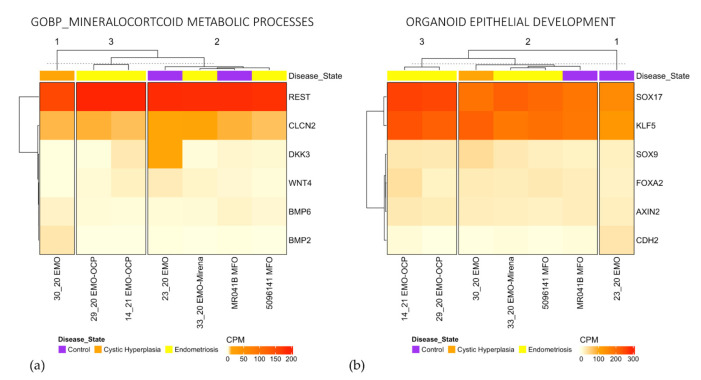
Gene expression heatmaps for Mineralocorticoid Metabolic Process (**a**) and Organoid Epithelial Development (**b**) show clustering of EMO-OCP but not EMO-H.

**Figure 7:**
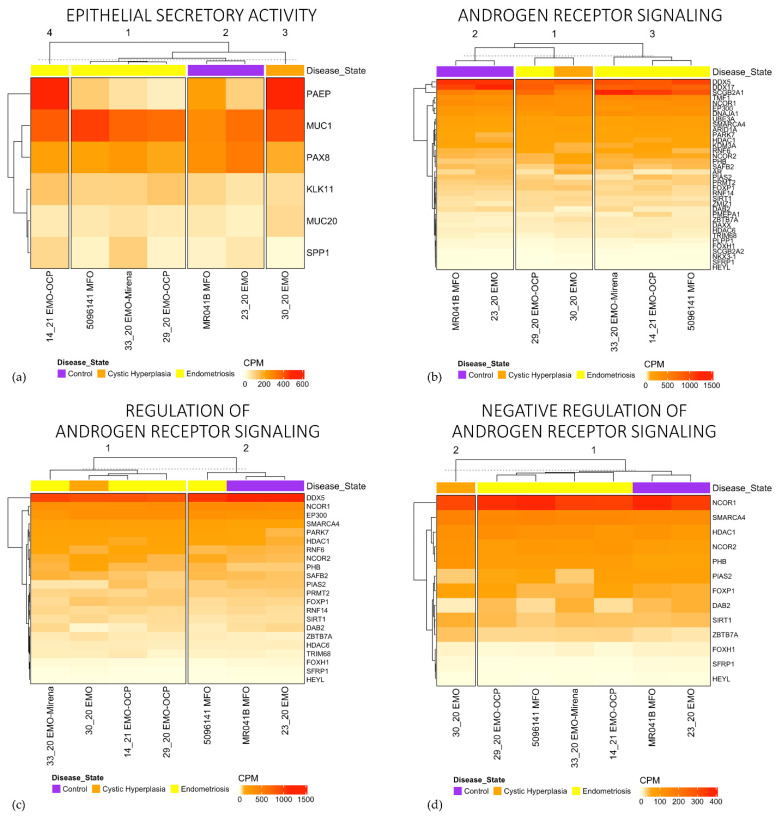
Gene expression heatmaps for epithelial secretory activity (**a**) and Androgen Receptor Signaling (**b**), and its regulation (**c**, **d**) shows clustering of controls from disease states.

While EMO-H rarely clustered separately from other organoid types, ccasionally EMO-OCP clustered together—for example, Response to Progesterone (Supplementary Figure S2a), Mineralocorticoid Metabolic Processes, and Organoid Epithelial Gland Development (Figure 6a,b). All of the EMO-H belonged to the same cluster for Response to Progesterone (Supplementary Figure S2a) and Regulation of Androgen Receptor Signaling (Figure 7c); however, this cluster also contained an EMO. There was no obvious clustering of MFO or EMO-H for any of the GO terms or curated sets that were analysed, indicating that for the small sample size that was analysed, MFO are similar to EMO and EMO-H, and EMO-H are similar to EMO and MFO.

### 3.5. Disease State Gene Expression Profiles

The individual gene expression profiles also clustered based on disease state for several gene sets. A number of GO gene sets as well as curated gene sets generated by us and others [22] showed the clustering of two control participants (23_20 and MR041B) compared to the other five participants. This included Epithelial Secretory Activity (Figure 7a) and Androgen Receptor Signalling and both the Regulation and Negative Regulation of Androgen Receptor Signalling (Figure 7b–d). These gene sets also showed the moderate clustering of endometriosis organoids, although at times, endometriosis clustered with cystic hyperplasia or one endometriosis participant clustered with controls (Regulation of Androgen Receptor Signaling). There was no association the between the proportion of EpCAM^+^ cells determined by flow cytometry (Figure 2b) and EpCAM mRNA levels (Supplementary Figure S2b).

## 4. Discussion

The main findings of our study are that organoids can be readily derived from menstrual fluid (MFO) and endometrial tissue from women who are taking hormonal medication (EMO-H) and can be cultured for multiple passages. The recent generation of endometrial organoids have generated a new, rapidly expanding field of research, resulting in 60 publications since endometrial epithelial organoids were first described in 2017. Endometrial organoids have already improved our understanding of endometrial biology [23,24], including interactions with the stromal niche [25], endometrial cancer [7], endometriosis [6], and polycystic ovarian syndrome [26]. Improvements in diagnosis and treatments for gynecological diseases are likely to be expedited by the study of early disease states from large numbers of women. Thus, in order to capture samples from such women, most of whom do not require an endometrial biopsy or who are currently taking hormonal medication, we have shown that organoids can be generated from menstrual fluid or from endometrial tissue from women taking hormonal medication. The efficiency of organoid generation was higher for EMO and EMO-H than it was for MFO. This may reflect both the quantity and quality of endometrial epithelial tissue that is available in menstrual fluid, where endometrial epithelial tissue fragments are less abundant and undergo tissue breakdown. MFO and EMO-H show similar rates of proliferation and endometrial progenitor cell surface marker expression to EMO.

We did note an effect of the type of hormonal medication on organoid characteristics. In particular, EMO-OCP tended to exhibit slightly greater rates of cell proliferation and a slightly lower percentage of EpCAM^+^ epithelial cells compared to all other organoids, including EMO-Mirena^®^. Apparent higher rates of cell proliferation in EMO-OCP are not unexpected, as OCP results in atrophic or inactive endometrium, similar to a menopausal state, which we have previously shown contains clonogenic epithelial progenitors [12] and N-Cadherin^+^ and SSEA-1^+^ epithelial cells in the basalis of hysterectomy tissue [3,27]. Therefore, it is likely that the proportion of clonogenic cells from OCP endometrium seeded into organoids will be higher compared to endometrium from women who are not taking hormones or from endometrial tissue fragments from menstrual fluid. In contrast, the proliferation rate of EMO-Mirena^®^ was not elevated despite progesterone-only contraception, which also causes atrophic or inactive endometrium. This may be explained by a lack of estrogen priming in progesterone-only Mirena^®^ compared to OCP [28] and the exposure of the epithelial cells to high levels of continuous progesterone. The estrogen priming of endometrial epithelial cells is important for endometrial epithelial proliferation [29]. Progesterone differentiates estrogen-primed, proliferating endometrial epithelial cells into non-cycling, histotroph-secreting cells [30,31].

The relevance of a reduced proportion of EpCAM^+^ cells in EMO-OCP (two of the four participants with endometriosis) is unclear and is worthy of further investigation. EpCAM regulates cell division—the extracellular domain (detected by flow cytometry) is cleaved by ADAM 10/17 and shed into the extracellular space. The intracellular domain is cleaved by γ-secretase and translocates into the nucleus and is together with adapters, and transcription factors bind the promoters and regulate the expression of the genes that are involved in cell division, pluripotency, and epithelial mesenchymal transition (reviewed in [32]). Thus, the lower proportion of EpCAM^+^ cells in EMO-OCP may indicate EpCAM cleavage and cell division.

Our method of generating MFO involves selecting EpCAM^+^ gland fragments by magnetic beading. A recent work [22] that was completed at the same time as ours confirms our initial observations that MFO can also be derived from unbeaded endometrial epithelial fragments from a small fraction of cells obtained from previous studies [9]. On the other hand, methods for standard EMO (and therefore EMO-H) generate EMO from fragments of unbeaded endometrial tissue that are separated by filtration and therefore may contain stromal tissue fragments or other cell types. There may be benefit in EpCAM beading to purify gland fragments for generating organoids from all of the different sources: endometrial tissue that has or has not been exposed to exogenous hormones or not and menstrual fluid.

Our data also showed variable proportions of cells that were positive for the surface marker SSEA-1 and for N-cadherin^+^SSEA-1^+^cells in some organoids. There were also variable mRNA levels of SSEA-1, which tended to be elevated in endometriosis, supporting our previous findings of SSEA-1 in functionalis endometrium [4].

Organoids that retain patient characteristics and that remain genetically stable represent potentially useful options for diagnosis as well as for understanding disease progression and response to treatments in individual women. Many women seeking diagnosis and treatment with endometrial pathologies are likely to be on hormonal medication for either the control of their symptoms or for birth control. Quality diagnostic and prognostic methods should not be influenced by these clinical variabilities, which would otherwise limit their utility. There is also a wide variety of hormonal compounds and concentrations that are taken by women that may have subtle effects. In this study, we show that the organoids derived from women under hormonal treatment are not significantly influenced in terms of the curated gene sets that are associated with the cellular targets of these hormones. This suggests the long-term utility of these in vitro models for characterizing patient response, as the organoids may recover their natural (basal) state once they are removed from the hormonal environment. This would ensure that potential diagnostic or prognostic biomarkers are not masked in an endometrium that has been rendered inactive through hormonal suppression.

There are a number of strengths of the current study. It is the first to demonstrate that organoids can be generated from both menstrual fluid and from the biopsies of women who are taking various forms of hormonal contraception. We show novel findings that compare the behavior and transcriptomes of EMO-H and MFO to standard EMO using well characterized samples. While organoids have been generated from eutopic and ectopic tissue (ECT-O) samples of women taking hormonal medication [6], that study did not compare different hormone types (e.g., OCP vs. progesterone only) on organoid characteristics. This is the first report indicating the potential clustering of EMO (as opposed to ECT-O) based on endometriosis disease state, a major advantage for minimally invasive precision medicine. There were some preliminary trends to indicate that endometrial organoids, regardless of their source (EMO, EMO-H, or MFO), may be used to distinguish controls from disease states. Further work to elucidate endometrium-specific and endometriosis curated gene sets may reveal previously unappreciated differences. In particular, distinguishing women with endometriosis from those without endometriosis using MFO would provide a non-invasive method of screening for a disease that currently depends on laparoscopic surgical diagnosis. Further work with increased power may reveal the endometriosis organoid subtypes that underscore clinical heterogeneity and that therefore may predict responses to new treatments that could be tested in vitro prior to application in vivo.

Conversely, the study is limited by a number of factors including low sample numbers due to the impact of COVID-19 on sample collection, permitted experiments, and delays in obtaining reagents. The low sample numbers and number of reads per sample limit the power of the study to truly detect any differences between either organoid source or disease state, and therefore, caution must be used in interpreting the findings. Further investigation in larger cohorts to determine the relevance of heterogeneity in organoid characteristics is warranted. Furthermore, future sufficiently powered studies that utilise EMO-H to determine how different clinically administered hormonal treatments influence both the short- and long-term health and function of endometrial tissue will provide powerful, clinically relevant data. MFO and EMO-H share similar features to EMO in proliferation, cellular composition, and gene expression in the limited number that was assessed.

## 5. Conclusions

This descriptive study highlights the potential for MFO and EMO-H as important tools for disease modelling and precision medicine. Novel organoids have the added advantage that MFO can be acquired non-invasively from menstrual fluid or EMO-H from large numbers of women who are already under hormonal treatment, respectively, including those with presumptive endometriosis without the need to cease hormonal medication, which remains the mainstay of the expectant management for endometriosis, heavy menstrual bleeding, and pelvic pain. Further work may establish if there are nuanced influences of different types of hormonal medication on endometrial epithelial behavior. The addition of the EpCAM^+^ selection of endometrial epithelial fragments in organoid isolation protocols may be used more widely to increase the purity of endometrial organoids and to reduce variation between studies. Together, MFO and EMO-H may widen the application of endometrial organoids for gynecological research and personalized medicine.

## Data Availability

The data presented in this study are openly available in Gene Expression Omnibus at https://www.ncbi.nlm.nih.gov/geo/ (accessed on 2 December 2021), accession number GSE188915.

## Supplementary Materials

The following are available online at https://www.mdpi.com/article/10.3390/jpm11121314/s1, Figure S1: EMO-H from women taking OCP (open green triangles) showed a trend for increased proliferation compared to EMO, MFO, and EMO-H from a woman with Mirena^®^ IUD, Figure S2: Gene expression heatmaps for response to progesterone (a) and epithelial markers of novel organoids (b), Table S1: Participant details and assays performed on each sample, Table S2: Flow cytometry reagents.
