# Supplementary material for: Comparison of Organoids from Menstrual Fluid and Hormone-Treated Endometrium: Novel Tools for Gynecological Research
Source: J Pers Med. 2021 Dec 6;11(12):1314. doi: 10.3390/jpm11121314 (PMC8707872; doi:10.3390/jpm11121314)
Supplement: Supplementary file 1 [file jpm-11-01314-s001.zip › jpm-1468449-supplementary.pdf]

Journal of Personalized Medicine

MDPI

Supplement

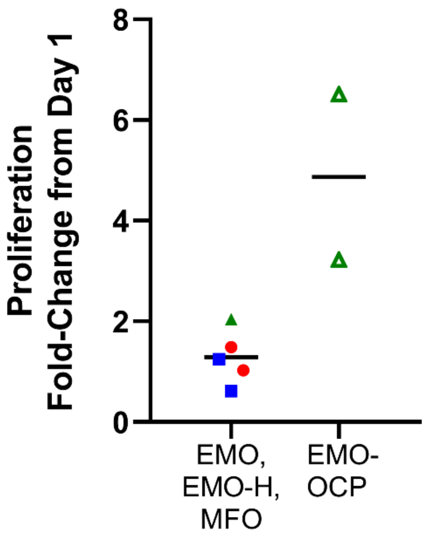

Supplementary Figure S1. EMO-H from women taking OCP (Open green triangles) showed a trend for increased in proliferation compared to EMO, MFO and EMO-H from a woman with Mirena® IUD.

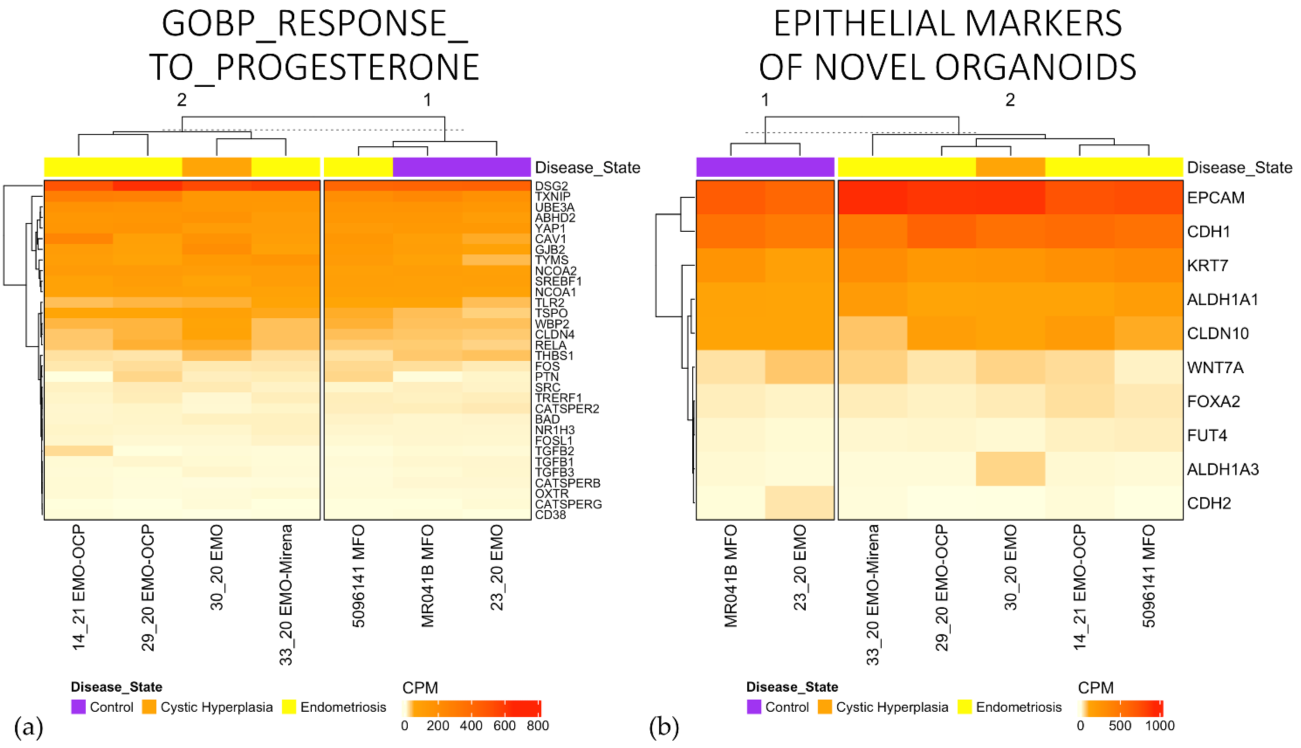

Supplementary Figure S2. Gene expression heatmaps for Response to Progesterone (a) and Epithelial Markers of Novel Organ-oids (b).

Journal of Personalized Medicine

MDPI

Supplementary Table S1. Participant details and assays performed on each sample.

ID	Organoid Type	Pathology	Tissue Type	Hormones	Age	Cycle Stage	Assays performed at Passage 3				
							EMO Images	Flow D7	Presto Blue D0,1,7	SSEA-1 IF	RNA seq.
MR041B	MFO	Population Control	MF	No	29	N/A	Y	Y	Y	Y	Y
5096141	MFO	Endometriosis; Stage n.d.	MF	No	34	N/A	Y	Y	Y	N	Y
29_20	EMO-OCP	Endometriosis; Stage IV	D&C	OCP	27	OCP (193 days since LMP)	Y	Y	Y	Y	Y
33_20	EMO-Mirena®	Endometriosis; Stage n.d., Chronic pain	Endo /Myo	Mirena®	35	Mirena® (53 days since LMP)	Y	Y	Y	Y	Y
14_21	EMO-OCP	Endometriosis; Stage n.d., Menorrhagia, Pain	D&C	OCP	42	Menstrual	Y	Y	Y	Y	Y
23_20	EMO	Menorrhagia	D&C	No	50	67 days since LMP	Y	Y	Y	N	Y
30_20	EMO	Menorrhagia, Cystic Hyperplasia	D&C	No	34	Proliferative	Y	Y	Y	Y	Y

Endometrial organoids, EMO; menstrual fluid organoids, MFO; EMO on hormones, EMO-H, EMO on oral contraceptive pill, EMO-OCP; EMO on Mirena®, EMO-Mirena®; dilatation and curettage, D&C; last menstrual period, LMP, n.d.; not determined.

Journal of *Personalized Medicine*

MDPI

Supplementary Table S2. Flow cytometry reagents.

Antibody	Manufacturer	Catalogue Number	Concentration
FcR Blocking Agent	Miltenyi Biotec, NRW DE	130-059-901	2 μL/10^6 cells*
IgG Blocking Agent	Jackson ImmunoResearch	012-000-002	4.4 μg/10^6 cells*
CD31-PeCy7	BD Biosciences, NJ, USA	563651	0.5 μL/10^6 cells*
CD45-PeCy7	BD Biosciences, NJ, USA	557748	0.5 μL/10^6 cells*
EpCAM-BB515	BD Biosciences, NJ, USA	565398	0.5 μL/10^6 cells*
N-cadherin-PE	BioLegend, CA, USA	350805	10 μg/mL
SSEA-1-BV421	BioLegend, CA, USA	125614	1.25 μg/mL
Propidium Iodide	BD Biosciences, NJ, USA	51-66211E	31.25 ng/mL

* Stock concentration not provided by manufacturer. EpCAM: Epithelial cell adhesion molecule; SSEA-1: Stage specific embryonic antigen-1.
